# Correlation of Gene Expression and Genome Mutation in Single B-Cells

**DOI:** 10.1371/journal.pone.0067624

**Published:** 2013-06-28

**Authors:** Joshua A. Weinstein, Xun Zeng, Yueh-Hsiu Chien, Stephen R. Quake

**Affiliations:** 1 Biophysics Program, Stanford University, Stanford, California, United States of America; 2 Department of Microbiology and Immunology, Stanford University, Stanford, California, United States of America; 3 Department of Bioengineering, Stanford University and Howard Hughes Medical Institute, Stanford, California, United States of America; University Magna Graecia, Italy

## Abstract

High-throughput measurement of gene-expression and immune receptor repertoires have recently become powerful tools in the study of adaptive immune response. However, despite their now-widespread use, both tend to discard cell identity by treating cell populations in bulk, and therefore lose the correlation between genetic variability and gene-expression at the single cell level. In order to recover this information, we developed a method to simultaneously measure gene expression profiles and genome mutations in single cells. We applied this method by quantifying the relationships between gene expression and antibody mutation in ensembles of individual B-cells from immunized mice. The results reveal correlations reflecting the manner in which information propagates between a B-cell’s antigen receptors, its gene expression, and its mutagenic machinery, and demonstrate the power of this approach to illuminate both heterogeneity and physiology in cell populations.

## Introduction

The mammalian adaptive immune system is comprised of T-cells and B-cells that produce receptors specific to antigens. For B-cells, these receptors, called immunoglobulins, or antibodies, form by the stochastic, genomic rearrangement of three alternate exons (V, D, and J) on a heavy chain and two exons (V and J) on a light chain. Random insertion and deletion of nucleotides between these exons during this process further potentiates enormous diversity. Antigen-engagement of antibody receptors on B-cell surfaces results in B-cell activation, up-regulation of the enzyme AID [1], and the consequent hypermutation of the antibody-encoding gene; the variants created by these mutations are yet another source of diversity. AID additionally induces antibody class-switching, whereby the non-mutated constant region of the antibody heavy chain gene, initially expressed as IgM and IgD classes, may change to IgG, IgA, or IgE. Because such diversification of antibody receptors, which fine-tunes adaptive immune response, both affects and is affected by the gene-expression of B-cells that produce them, co-variation between receptor sequence and immune gene-expression may be expected to reflect direct and indirect mechanisms of feedback between them. While high-throughput measurements have examined both independently in bulk samples [2], [3], [4], [5], no combined cell-to-cell analysis of these two critical components of immune response has yet been performed.

## Materials and Methods

BALB/c mice were purchased from the Jackson Laboratories. TCRδ−/− mice (on BALB/c background) [6] were bred in the Stanford Animal Facility. BALB/c and TCRδ−/− mice were housed together in the same cage for at least a week before immunization. All experiments were approved by the Administrative Panel on Biosafety and the Administrative Panel on Laboratory Animal Care at Stanford University (Permit Number: 9456). The mice were sacrificed in a carbon dioxide container and all efforts were made to minimize suffering.

**Figure 1:**
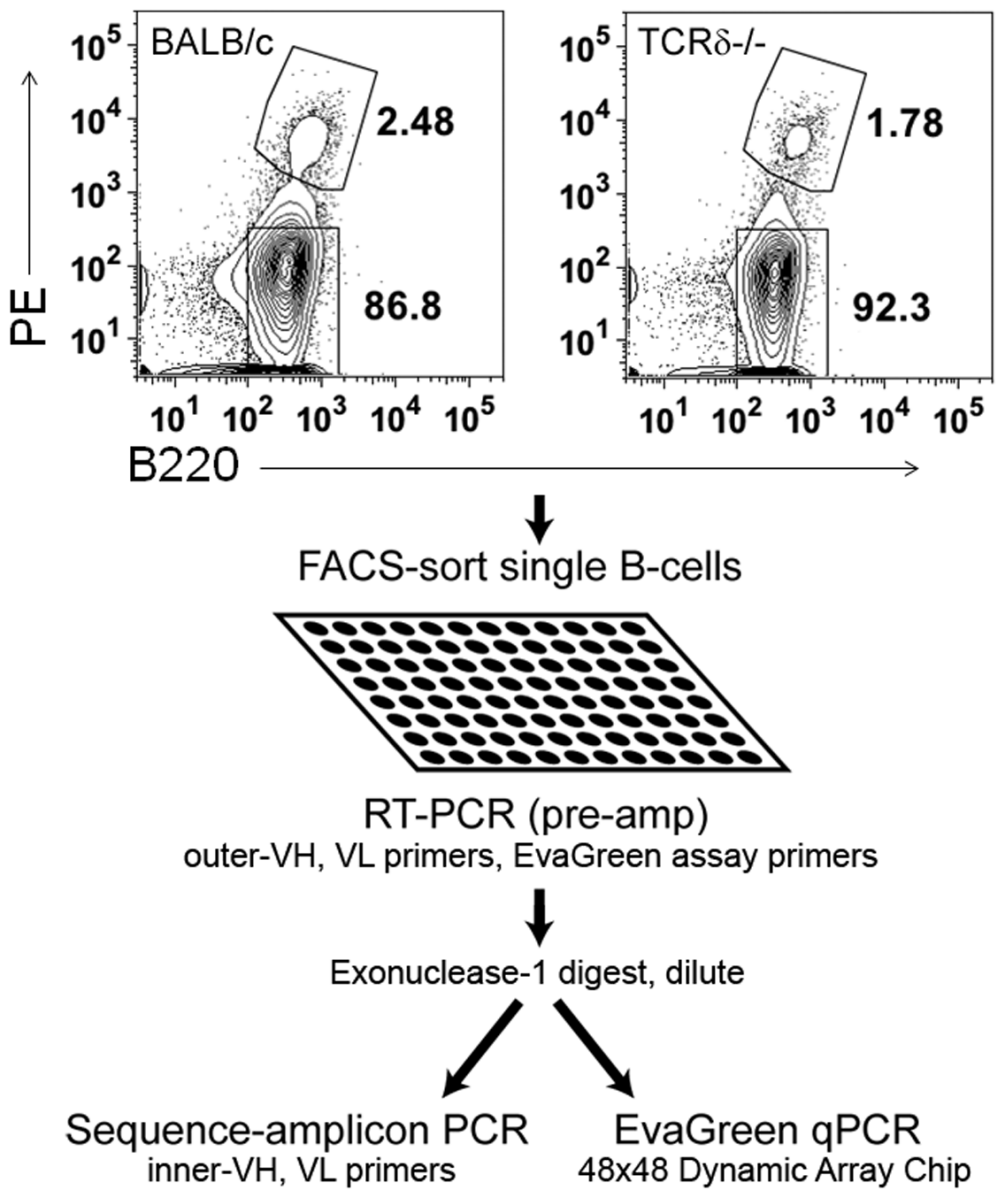
Experimental workflow. Single PE+/B220+ and PE−/B220+ B-cells were FACS-sorted into 96 well plates, pre-amplified using a pool of gene-expression and sequencing-amplicon outer-primers for heavy- and light-chains (see Tables S4 and S5 in File S1). After exonuclease digestion of leftover single-stranded primer, PCR products were split between sequencing-amplicon amplification using inner-primers and quantitative PCR using EvaGreen dye on Fluidigm 48×48 Dynamic Array chips.

We investigated the statistical relationships between Ig sequences and the gene-expression programs of B-cells producing them. One BALB/c mouse and one TCRδ−/− mouse, which lacked γδ T cells were immunized with phycoerythrin (PE). Although αβT cells are necessary for the generation of germinal center B cell response, γδ T cells can recognize the same antigens as B-cells and thus may affect B cell development [7]. Fourteen days after immunization, mice were sacrificed, draining lymph nodes dissociated, and cells stained for PE-binding (Text S1 in File S1). Single PE+ and PE- B-cells were sorted and pre-amplified with primers specific both to sequences flanking the variable regions of the Ig heavy- and light-chains (Figure 1, Table S4 in File S1) and a panel of genes noted for their expression in differentiating B cells (Table S5 in File S1). Quantitative RT-PCR was performed on Fluidigm 48×48 Dynamic Array microfluidic chips using EvaGreen dye and antibody heavy- and light-chains were Sanger-sequenced (Text S1 in File S1). 368 cells were sorted, and 193 passed gene-expression and sequence quality-filters for use in further analysis (Text S2 in File S1, Text S3 in File S1).

The gene panel was chosen to investigate several aspects of B-cell state, including differentiation, activation, and proliferation (Table S6 in File S1). GAPDH, HSP90, HPRT, and GUSB were included to provide information on cellular metabolism, and CDKN1A and HDAC5 for information on cell cycle. AID was included, as were all antibody isotypes (IgA, IgD, IgE, IgG, and IgM), with IgG subdivided into three subtypes, IGHG1, IGHG2B, and IGHG2A/C. We included CD22, CD79A, IGBP1, FCGR2B, FCER2A, FCAMR, CR2, CD19, PI(3)K, (coded for by the PIK3CD gene), DOCK8 and CD40, associated with trans-membrane signaling by antibodies [8], [9], [10], EBI-2 and LTA, involved in B-cell migration and lymph node and germinal center organization, respectively [9], [10], [11], [12], and GNAI2, involved in B-cell motility [13]. We further included PRDM1 (or BLIMP-1), IRF4, and BCL6, the former two involved in B-cell terminal differentiation into plasma cells and the latter involved in a B-cell’s persistence in the germinal center [9]. Also included were pro- and anti-apoptotic genes (BAD and MCL-1 [14], respectively), genes involved in curtailment of hyperproliferation and autoimmunity (IL-10 and TNFRSF13B [15], [16]), and protein kinase C-family members involved in activation and self-tolerance (PRKCB and PRKCD, [17]). Several other genes previously found associated with B-cell activation and differentiation (CD5, CD81, MS4A1 or CD20, CLCF1, PTPRC, IL-12, TNFRSF8, TNFSF8, TNFRSF13C or BAFF-R, and SLA-2) were also included [18], [19], [20], [21], [22], [23], [24], [25], [26]. RAG1, responsible for antibody recombination during B-cell development, but not expressed by mature B-cells, was included as a negative control.

## Results and Discussion

**Figure 2:**
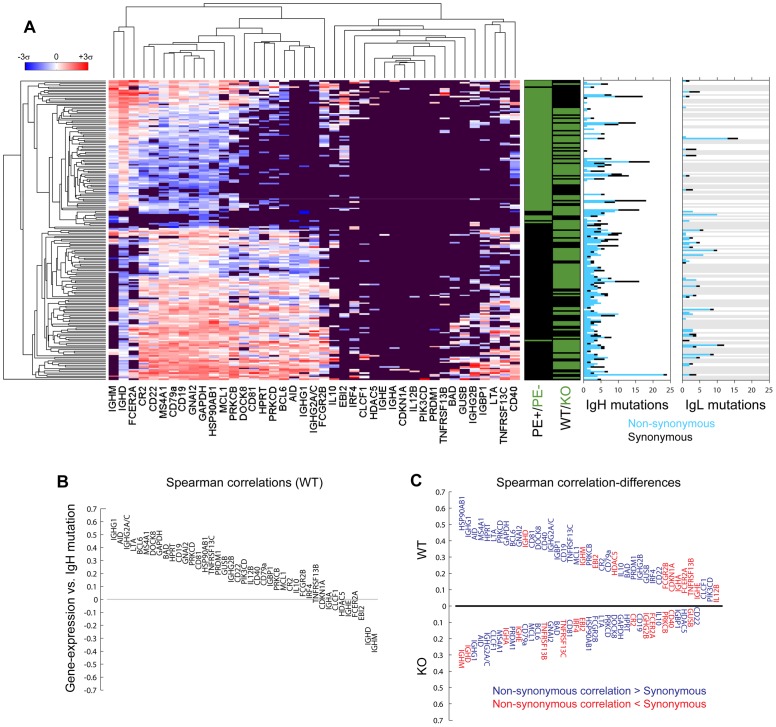
Interrelations between antibody mutations and immunological gene-expression in single B-cells. Normalized gene expression values (red denotes up-regulation and blue denotes down-regulation) were hierarchically-clustered across 193 single PE+ and PE- B-cells belonging to BALB/c (WT) and TCRd−/− (KO) mice, and plotted alongside mutational content of antibody heavy- and light-chains expressed by each (**A**). For the latter, cells for which light-chains either could not be sequenced or were rejected by the quality-filter are color-coded on the bar-plot in grey. Spearman-correlations were calculated between each gene-expression value and mutation-count for wild-type (**B**) and knock-out (Figure S3A in File S1) mice. Data were further analyzed by calculating differences between Spearman correlations performed on non-synonymous and synonymous-mutations separately (**C**), with absolute values of these differences depicted on the vertical axis.

We analyzed gene-expression patterns and found these clustered almost entirely by B-cell phenotype (Figure 2A). Principal component analysis revealed that the first and second principal components (Figure S2 in File S1) captured 56% of total variance and classified PE- and PE+ B-cells, respectively, with 94% accuracy. No such classification was observed for mouse-type. This demonstrated that intrinsic variation of gene expression between cell types dominated over the extrinsic variation of gene expression between animals.

We began our antibody sequence analysis by focusing on the antibody heavy chain, both because it bears the greatest responsibility for antibody specificity in general [27] and because its large VDJ-junctional region avails it of far greater diversity as compared to the light chain. Moreover, this extended junctional diversity, generated randomly from somatic gene recombination undergone during B-cell development, provides crucial information about the common ancestry of B-cells with the same V/J recombination. This information can be used to illuminate the processes of B-cell development and clonal expansion in data-sets of arbitrary size [5]. We classified antibody sequences according to clonal lineage by performing single-linkage clustering on their heavy chain CDR3-regions, with a distance-threshold of one amino acid, and found evidence for clonal expansion exclusively among PE+ B-cells (Figure S1 in File S1). This helped to confirm that no systematic cross-contamination had occurred between cell samples. Using these heavy-chain clonal lineage designations as a benchmark, light-chains that had been successfully sequenced were filtered according to a statistical test that quantified the likelihood of a heavy/light-chain pairing to have occurred randomly, in order to eliminate potential PCR-contaminants (Text S4 in File S1). This further helped correct for B cell clonal lineages that had been mis-assigned as distinct from one another based on heavy chain sequence alone (Figure S5 in File S1). We also analyzed our raw data directly, without using light-chain sequence-filtering or correction of antibody clonal lineages, and observed qualitatively identical results (Figures S4, S6, S7 in File S1, Tables S1, S2, S3 in File S1). The B-cell lineages originated in several distinct V/J-combinations (Figure S1 in File S1). However, no overlap among clonal lineages, as characterized by their heavy-chain CDR3 regions, was found between PE+ and PE- populations. We next analyzed antibody gene mutational content by tallying synonymous and non-synonymous mutations for each cell’s heavy and light chains. We found that whether a light chain had at least one mutation strongly depended on whether its heavy chain had at least one as well, with p<0.004 (one-tailed Fisher’s Exact Test) for all mutations together and with p<0.005 for non-synonymous mutations alone (Table S1 in File S1).

### Correlations between Antibody Somatic Hypermutation and B-cell Gene Expression

We compared antibody mutations from each cell to determine how the same cells clustered by global patterns in gene expression (Figure 2A). Surprisingly, while virtually all un-mutated antibody heavy- and light-chains clustered within the IgM+/IgD+ population, so too did many of the most mutated antibodies, contravening the widespread notion that such antibodies would only be that way due to antigen-specific selection. Heavy-chain mutations averaged 4.5±3.2 and 2.6±4.7 per cell for PE+ and PE- fractions, respectively, and light-chain mutations averaged 3.5±3.0 and 1.1±2.8, respectively (similarly high variation was observed for each cell type when non-synonymous and synonymous mutations were considered independently). Therefore, the average differences between the two cell populations were swamped out by variability within them.

Proceeding across the entire data-set, we calculated the Spearman correlations between each gene-expression value and the heavy-chain mutations accumulated by the corresponding cell. Strikingly, AID was the gene most positively correlating with mutations in the TCRδ−/− mouse (Figure S3A in File S1) and the second most positively mutation-correlating gene in the BALB/c wild-type mouse (Figure 2B). This result demonstrated that the gene most directly and mechanistically responsible for somatic antibody mutations is also the gene whose expression was most informative about their accumulation.

The isotype correlation and anti-correlation with somatic mutation were consistent with the standard model for B cell maturation. IgG expression, requiring AID-induced isotype class-switching, correlated positively with somatic mutation. Conversely, IgM and IgD correlated negatively with somatic mutation, affirming their preferential expression by un-activated, non-mutating B-cells. Such negative correlations, found in both mice, existed for only a few other genes. The most prominent of these were EBI-2 (or G-protein coupled receptor 183), expressed on plasma and non-germinal center B-cells [9], [10], and the FCER2A receptor (CD23), responsible for the membrane-display of IgE antibodies on B-cells. The latter gene, clustering with IgM’s and IgD’s expression pattern more generally (Figure 2A) was especially surprising: FCER2A’s up-regulation might have been expected most on B-cells undergoing class-switching of antibody isotypes, and therefore activation. However, the relationships between FCER2A and EBI-2 made clear how the opposite effect may arise. FCER2A is specifically up-regulated in response to EBI-2 expression [28], and meanwhile, EBI-2 expression is actively down-regulated among germinal center B-cells [9], the B-cells most actively undergoing hyper-proliferation and hypermutation. The anti-correlation observed between FCER2A expression and somatic mutation is therefore able to emerge from an indirect, but distinctly negative, relationship between the two.

### Independent Sampling of Antibody Lineages Eliminates Clonal Bias

The correlations so far discussed were calculated across individual cells. However, one could imagine that clonal relationships might bias results, especially if phenotypic state is inherited during cell division. In order to eliminate this possibility, we used our knowledge of the B-cell population’s clonal relationships from their antibody sequences. By iteratively and randomly sampling cells from each clonal lineage, we ensured each would be equally represented in the final, averaged, correlation calculation. The resulting correlation values were qualitatively the same as those performed across cells individually (Figures S6, S7 in File S1). Clonal relatedness did not therefore play a significant part in defining the observed gene expression-mutation correlations. These relationships were therefore found to represent independently-sampled gene expression programs.

Iterative, independent sampling of cells across each clonal antibody lineage allowed us to calculate Spearman rank-permutation p-values across the data-set for each mutation/gene-expression relationship (Tables S2, S3 in File S1). Broad significance was observed among the genes correlating most positively and most negatively with somatic mutation. Genes showing strong statistical significance for both mice independently consisted of AID, the IgG and IgD antibody isotypes, and DOCK8. DOCK8, correlating positively with mutation (p<0.02 in BALB/c and p<0.007 in TCRδ−/−), is a critical member of the pathway conveying information about antibody-antigen binding [14], [16] and thereby promotes high-affinity antibody production [19]. Its consistently significant correlation with mutations may suggest an active strengthening of a B-cell’s ability to check the efficacy of mutagenesis that is coincident with the accumulation of the mutations themselves.

### Effect of Mutation-type on Mutation/gene-expression Correlation

We next investigated the degree to which antibody-mutation/gene-expression correlations depended on the nature of the mutations themselves (sampling by cells in Figure 2C, sampling by clonal lineage in Figures S6C and S7C in File S1). In both mice, AID and IgG1 ranked in the top three genes for which correlations with non-synonymous mutations exceeded correlations with synonymous mutations. This suggested their regulation was among the most strongly coupled to antibody protein-diversification. Surprisingly, however, the magnitudes of the differences between non-synonymous and synonymous mutation/gene-expression correlations were very different between the two mice (asymmetry in Figure 2C).This stood in sharp contrast to the similarity between the magnitudes of mutation/gene-expression correlations themselves (symmetry in Figure S3B in File S1).

The fact that non-synonymous and synonymous mutation-correlations were more similar in the TCRδ−/− mouse may indicate a greater indifference, in general, by its gene-expression program to changes in antibody-antigen affinity. Supporting this interpretation was the fact that BAD, a pro-apoptotic gene, correlated more positively with antibody mutation in BALB/c B-cells (p<0.009, Table S2 in File S1) than the anti-apoptotic gene MCL-1 (p<0.8), whereas in the TCRδ−/− mouse, the opposite was true. These observations, together with the fewness of its expanding clonal lineages (Figures S1, S5 in File S1), suggested a greater degree of negative feedback experienced by B-cells during hyper-proliferation in the BALB/c mouse as compared to that in the TCRδ−/− mouse.

### Conclusions

The adaptive immune system selects B-cells that produce high-specificity antibodies to target a wide range of pathogens. Active feedback between induced antibody mutation and its effect on antibody specificity is therefore at the core of a successful immune response. In this paper, we measured this feedback by performing simultaneous measurement of gene-expression and antibody-gene variation across ensembles of individual B-cells, thereby quantifying the co-variance between these two sets of variables. This method provides information on how B-cell sensory and response mechanisms couple to and change each other. Its capacity to do so makes the method a powerful tool for answering emerging questions about the role of B-cells in regulating auto-immunity [29], and able to clarify the divergent behaviors of B-cell sub-populations. It may furthermore strengthen existing strategies for monoclonal antibody therapy development, by linking sets of antibody mutations with the up- and down-regulation of genes associated with antigen-binding. Taken together, our results demonstrate the power of this simultaneous gene-expression and mutation measurement to elucidate statistical relationships and heterogeneity otherwise hidden from studies that treat such cell-populations in bulk.

## Supporting Information

File S1 (DOCX)
