# Supplementary material for: Correlation of Gene Expression and Genome Mutation in Single B-Cells
Source: PLoS One. 2013 Jun 28;8(6):e67624. doi: 10.1371/journal.pone.0067624 (PMC3695916; doi:10.1371/journal.pone.0067624)
Supplement: File S1 — (DOCX) [file pone.0067624.s001.docx]

**Supporting Information**

Text S1: *Materials and methods*

One BALB/c mouse and one TCRd-/- (BALB/c background) mouse were subcutaneously immunized with PE/Alum (100 ug per mouse) at the tail base. After 14 days, cells from draining lymph nodes (inguinal) were stained with Aqua live/dead, Pacific Blue conjugated CD3, H57, CD11b, CD11c,Ter119, Gr-1,F4/80, PE (100ug/ml), APC-Cy7 conjugated anti-IgD, eFluor605 conjugated B220 1hr on ice. Aqua-,Pacific Blue-,B220+, PE+ cells or Aqua-,Pacific blue-,B220+, PE- cells were sorted into 5 ul master mix containing 2x reaction mix (from CellsDirect One-Step qRT-PCR Kit, Invitrogen 11753) and 0.1 ul SUPERase-In RNase Inhibitor (Ambion, AM2696). The first two and last two wells of each plate were left blank for positive- and negative-controls. Cells were immediately frozen down at -80 degrees C.

To thaw, plates were spun down at 4 degrees C for 5 minutes at 250xg. Into each well of 5.1 ul cell-lysate, were added 0.2 ul Superscript III RT-Taq Mix (Invitrogen 11753), 1.3 ul nuclease-free water, and 2.5 ul pre-amp mix, in TE, containing 200 nM of each DELTAgene-primer pair, 1.2 uM of VHF-1 primer-mix, 1.6 uM of VLF-1 primer-mix, 1 uM of VHR-seq primer-mix, and 0.4 uM of VLR-seq primer-mix. This brought the final volume in each reaction to 9 ul. To positive-control wells, 1 ul of single-use aliquot splenic total RNA from Balb/C mice (Agilent 736019) at 9 nmol/ul, was added.

Plates were vortexed 15 s and spun down for 2 minutes at 250xg. Samples were thermocycled 50 degrees C for 15 minutes, 95 degrees C 2 minutes, and 20x(95 degrees C 15 s, 60 degrees C 4 minutes). Afterward, 4 ul of Exonuclease-1 mix (New England Biolabs, M0293), containing 20% Exonuclease 1 at 20 units/ul, 1x reaction buffer, and 70% water, was added, vortexed, and spun down. This was then thermocycled for 30 minutes at 37 degrees C, followed by 15 minutes at 80 degrees C. 14 ul products were diluted 1:5 in TekNova DNA suspension buffer (TEKnova T0221) and frozen down at -20 degrees C.

After pre-amplification and Exo-1 treatment, diluted samples were thawed. Heavy- and light-chain 4x primer mixes were prepared, containing either 6.8 uM inVH mix and 4 uM VHR-seq mix, or 8.8 uM inVL mix and 1.6 uM VLR-seq mix, in TE. For each cell-reaction 2.5 ul of each of these were added to separate master-mixes containing 12.25 ul of nuclease-free water, 2.5 ul HiFi reaction buffer (Invitrogen 11304029), 1 ul 50 mM MgSO_4_, 0.5 ul 10 mM dNTP (Invitrogen 18427088), 1.25 ul DMSO (Sigma-Aldrich D9170), and 0.25 ul HiFi Platinum Taq (Invitrogen 11304029). To these 24 ul mixes, 1 ul of sample was added. After vortexing briefly and spinning down at 4 degrees C, plates were thermocycled 95 degrees C for 2 minutes, followed by 10x(94 degrees C 1 minute, 62 degrees C 1 minute, 72 degrees C 1.5 minutes), with the temperature 62 degrees C decreasing by 1 degree Celsius every cycle to 52 degrees C. An additional 30 cycles of 94 degrees C 1 min, 52 degrees C 1 min, 72 degrees C 1.5 min were then performed, followed by a final extension of 72 degrees C for 10 minutes. PCR-products observable on a gel between 400-500 bp were Sanger-sequenced (Sequetech, Mountain View, CA) using VHR-seq (IgD excluded in order to avoid incoherent signals from IgM/IgD co-expression) or VLR-seq primers (Table S4).

Fluidigm chips were run according to manufacturer’s specifications for single cell EvaGreen quantitative PCR assays (Fluidigm protocol ADP 30). All 48x48 chips were run on a single Fluidigm Biomark with Data Collection software v. 3.0.2.

Text S2: *Quantitative PCR analysis*

Fluidigm chip-readouts were normalized to the median-C_t_ readouts of their positive controls (each taken from the same total RNA stock solution). Dilution-curves (comprising total RNA amounts 9000 pg, 900 pg, 90 pg, 20 pg, and 4 pg) showed increasing noise in C_t_ values above a value of 20. Therefore, 20 was set as the maximum-positive C_t_. Cells having GAPDH C_t_’s falling above this value were discarded. Purple-colored reactions in Figure 1A of the main text and Figure S4 represent reactions with C_t_’s above this maximum value (or those with a gene-expression value, or –C_t_, falling ≥3σ below the mean *positive* gene-expression value for that assay).

We used the amplicon melting curve data to reduce noise in C_t_. Comparing the outputted “peak-ratio” parameter, *r*, generated by Fluidigm’s Biomark analysis software for each EvaGreen reaction to the high-Tm (good product fluorescence, *g*) and low-Tm (bad product fluorescence, *b*) peaks on individual melting curves, we found the empirical relation *b/g = (1.4-r)/(0.26+r)*. We used this to correct C_t_ values by considering each C_t_ value as proportional to log_2_ of the template amount, giving new C_t_ values *C_t_’ = C_t_ + log_2_(1+b/g)*. “Perfect” peak-ratios of 1 or “failed” peak-ratios of 0 were nevertheless left un-corrected or treated as negatives, respectively. The effect of melting-curve correction was measured both by measuring the average change in dilution-slopes for C_t_-vs.-log_2_ concentration scatter plots (perfect if equal to -1) and the average positive/negative accuracy (fraction true-positive or true-negative out of all positive and negative controls). Before C_t_-correction, these values were -0.72±0.28 and 91%±17%, respectively. After C_t_-correction, they improved to -0.75±0.27 and 96%±11%, respectively.^[[1]](#footnote-1)^

**
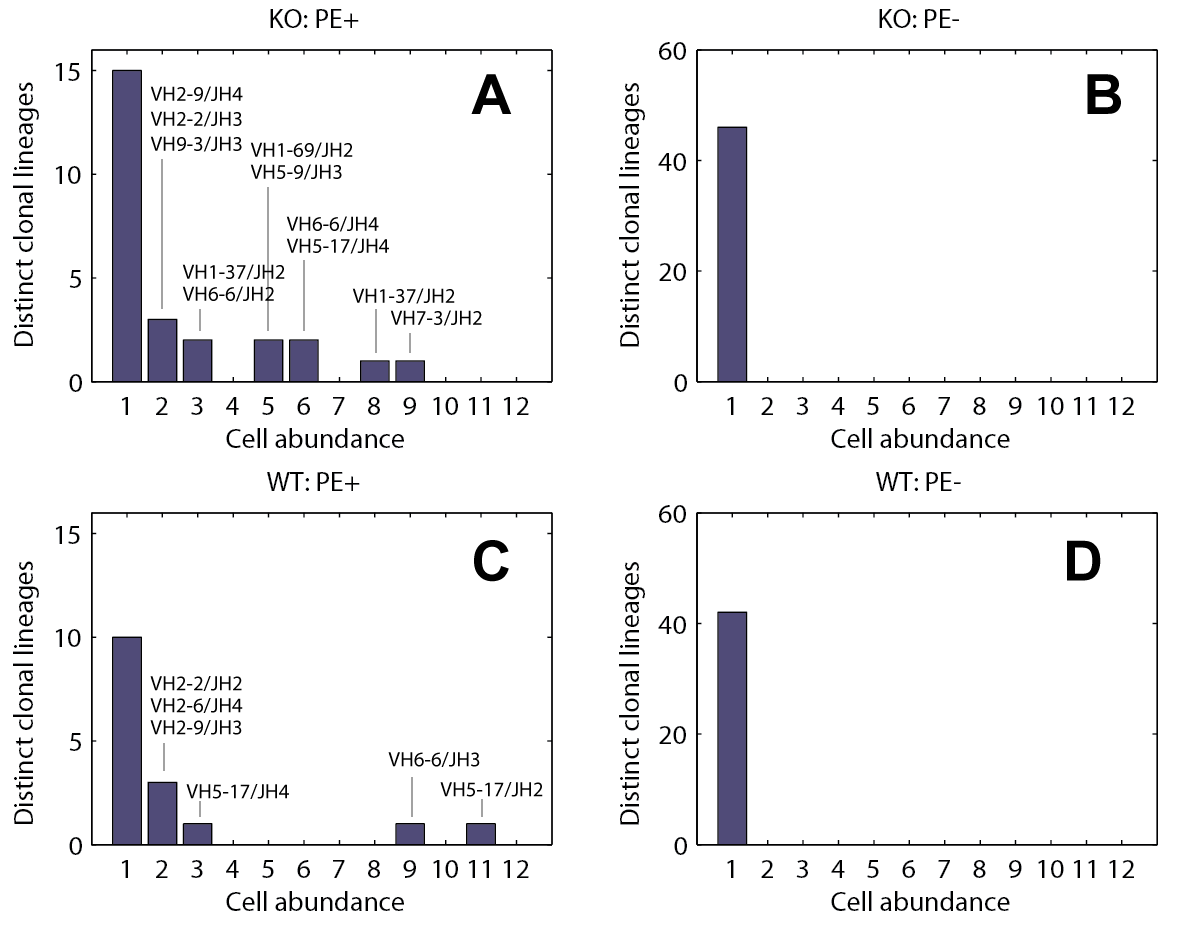
**

**Figure S1: Clonotype abundances:** Antibody sequences clustered by clonal lineage type identified by heavy-chain CDR3 identity (see *Antibody sequence analysis*). Evidence for clonal expansion is found among PE+ cells exclusively.

**
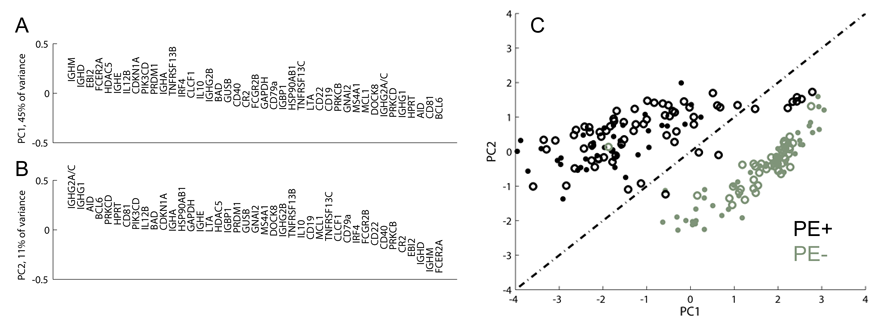
**

**Figure S2: Principal components and cell-partitioning.** PCA of data depicted in Figure 1 of the main text shows the first principal component (**A**), with 45% of total variance, holding the highest values among pre-activation genes, with IgM and IgD both topping the list. The second principal component (**B**), with 11% of total variance, holds the highest values for post-activation genes, with IgG and AID topping the list. Cell-types partition roughly accordingly (**C**, dots denote wild-type and circles denote TCRd-/-), with 87 out of 88 PE- B-cells having a higher value along PC1 and 95 out of 105 PE+ B-cells having a higher value along PC2. On average, this gives a 94.3% correct partitioning.

Text S3: *Antibody sequence analysis*

Source code, along with data for this study, are available for download at http://sourceforge.net/projects/ighanalysis/files/singleCell/

Sequences were analyzed by Smith-Waterman alignments to mouse IMGT reference sequences using software previously described (Jiang et al 2011). Cells were rejected if they had an average Sanger PHRED-score (defined as -log_10_ of the probability of being correct) less than 25, an unidentifiable CDR3 sequence (flanked by ta(t/c)t(a/t)(c/t)tg(t/c) to the 5’ bound and the best alignment of ta(t/c)tgggg to the 3’ bound as per Kabat et al 1991), or one with ambiguous nucleotides. Of the 368 cells sorted, 193 passed this and the qPCR filter discussed earlier for further analysis.

For light-chains, the same was done, only replacing the 3’-bound alignment with ttcgg(a/t/c)(g/t/a)(g/c)(g/t/a). Codon-mutations (non-indels only) were counted, using the CDR3 frame for reference, across the J-region downstream from the CDR3 region and across the V-region upstream from the CDR3 region for 220 bp. Mutations from base-calls with PHRED-scores less than 10 were ignored and those with PHRED-scores of at least 30 were given full weight. PHRED-scores between the two values were used to scale the weights linearly (eg a mutation with PHRED score of 20 would receive weight ½). Discrete mutation values, such as those illustrated in Figure 2 of the main text, Figure S4, and Table S1 are rounded from the PHRED-weighted values.

Text S4: *Light chain analysis*

Because of the far greater potential diversity of heavy-chain rearrangements (due both to the presence of a D-segment and the greater number of N-nucleotides), heavy-chain sequences provide the best information regarding the common ancestry of B-cells. Clonotype analysis (Figure S1) was performed by single-linkage clustering of sequences with identical VH- and JH-segment matches, such that two sequences possessing CDR3 sequences differing by at most 1 amino acid would cluster together. By doing this, we took advantage of the randomized deletion and insertion of N-nucleotides at the interface between VH, DH, and JH-exons. The total absence of evidence for clonal expansion among both antigen-negative cell populations helped to confirm that no systematic cross-contamination had occurred, and among VH-amplicons, no external contamination either. Nevertheless, the sensitivity of combined pre-amplification and post-amplification on sequence-products resulted in the failure of 2 out of 8 light-chain negative controls (1 out of 2 for PE-/KO and 1 out of 2 for PE+/WT). Sequencing the resulting false-positive product showed it bore resemblance to the mixed-signal obtained from total splenic RNA positive controls.

Eliminating potential light-chain contamination events on the basis of similarity between those belonging to unique heavy-chains (or heavy-chain clonal lineages) is non-trivial, because of the reduced diversity mentioned above (light chains lack a D-segment, and many of the light chains sequenced had few or no N-nucleotides at all between their VL and JL exons). Therefore, to establish confidence in each VH- and VL-pairing, a stringent statistical test was applied to accept or reject light-chain sequences. For each cell population, P-values for each light-chain clonal lineage were calculated based on their enrichment within each heavy-chain lineage using Fisher’s Exact Test:


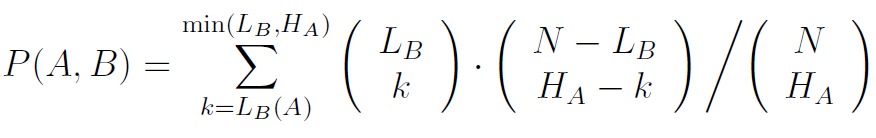


where L_B_(A) is the number of cells corresponding to the light-chain (B)/heavy-chain (A) pairing of interest, H_A_ is the total number of cells possessing the heavy-chain of interest, L_B_ is the total number of cells possessing the light chain of interest, and N is the total number of cells in the population.

Heavy-chain lineages were iteratively allowed to merge if and only if they would cluster based on a relaxed criterion (allowing 2 amino acids to differ at their CDR3 region, and their V- and J-segments to belong to the same exon-families, as opposed to sub-families) and the resulting number of cells in the population possessing P-values less than 0.03 would be maximally increased. Ties were broken by whichever cluster-merger resulted in the least diversity of light chain-lineages within the newly formed heavy-chain lineage-cluster. This algorithm would terminate once no VH-lineage merger could keep constant or increase the total number of cells with light-chain P-values less than 0.03. All light chain sequences having P-values at least 0.03, or sharing a heavy-chain clonal lineage with a light chain sequence of smaller P-value, were rejected.

The resulting set of light-chain sequences successfully excluded all instances of the detected contaminant, and merged several existing clonal lineages. As before, evidence for clonal expansion remained exclusively confined to the PE+ populations – as shown in Figure S5. More generally, this light-chain correction of antibody clonal lineages did not qualitatively change any of the observed gene-expression/mutation correlations that incorporated lineage-identity (Figures S6, S7 and Tables S4, S5) or the correspondences between heavy- and light-chain mutation (Table S1).


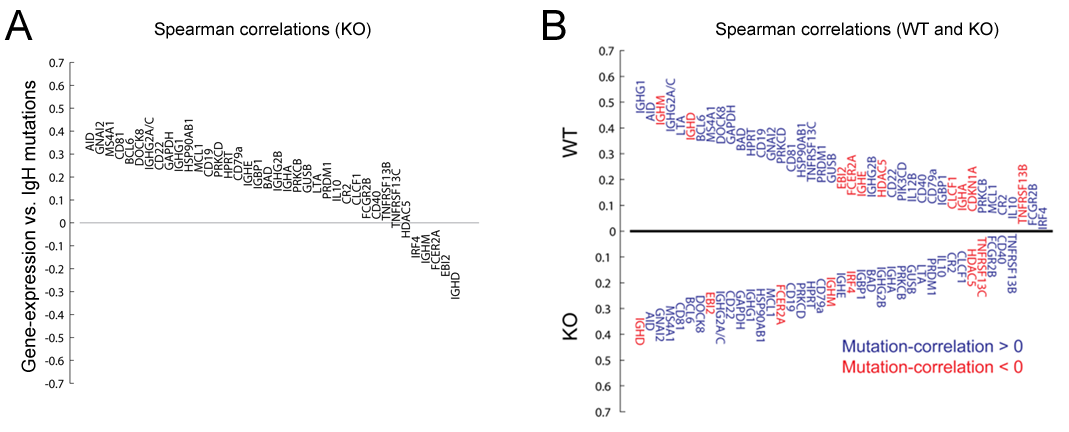


**Figure S3:** Spearman-correlations, in the same manner as main text Figure 2B, were calculated between each gene-expression and heavy-chain mutation-count belonging to each individual cell measured in the TCRd-/- mouse (**A**). These were depicted alongside wild-type correlations (from main text Figure 2C) by plotting absolute values along the vertical axis (**B**) and color-coding according to correlation-sign.

**
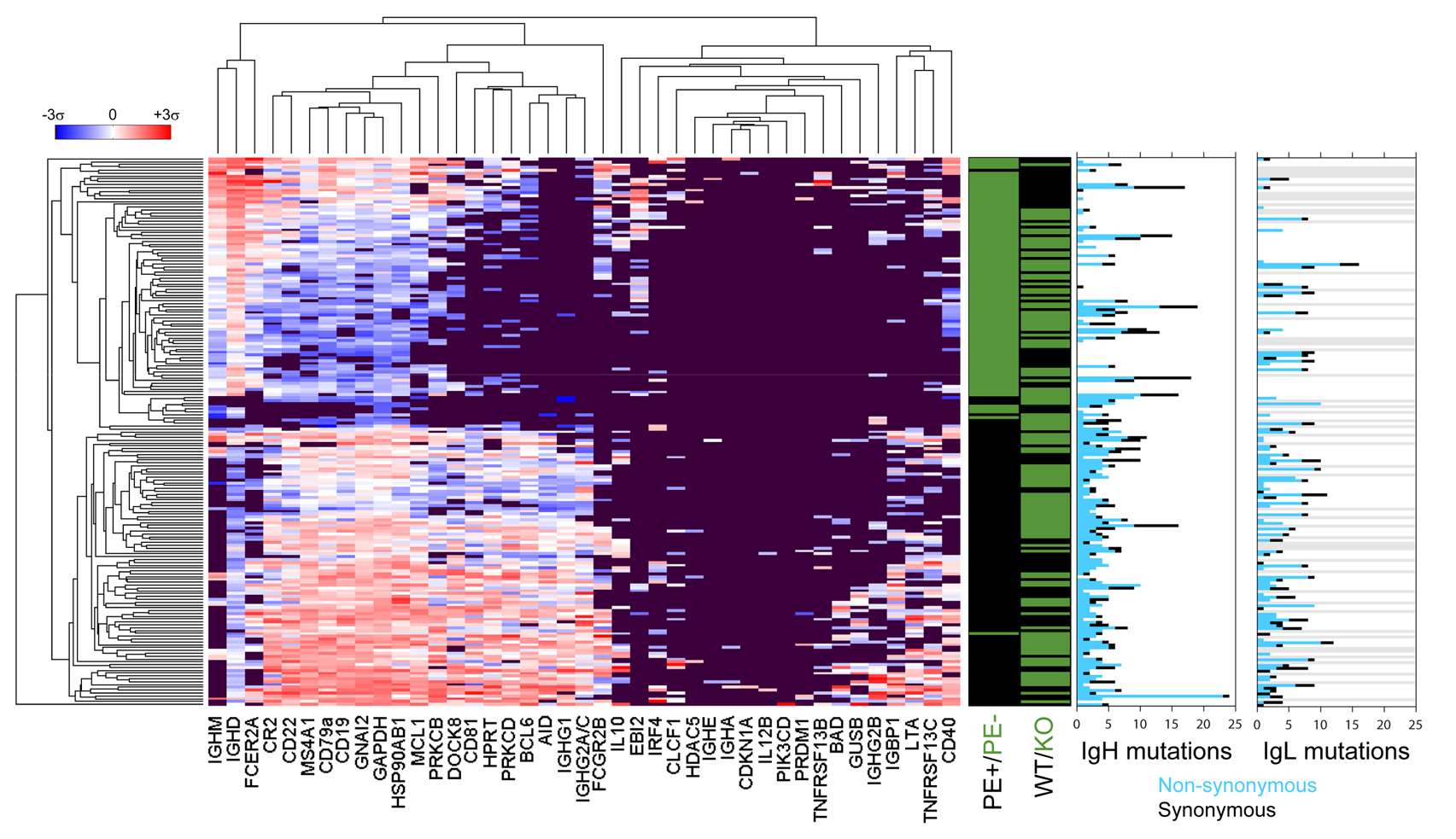
Figure S4: Clustered gene-expression/mutation data.** Data are illustrated in the same manner as main text Figure 2, without stringent filtering of light-chain sequences (described under *Light chain analysis*). Normalized gene expression values (red denotes up-regulation and blue denotes down-regulation) were hierarchically-clustered across 193 single PE+ and PE- B-cells belonging to BALB/c (WT) and TCRd-/- (KO) mice, and plotted alongside mutational content of antibody heavy- and light-chains expressed by each. For the latter, cells for which light-chains could not be sequenced are color-coded on the bar-plot in grey.


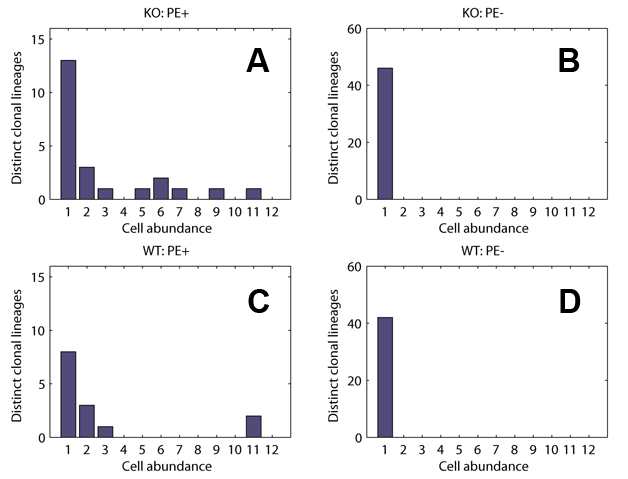


**Figure S5: Clonotype abundances, corrected by light-chain identity.** Clonal lineages calculated under the heavy-chain CDR3-similarity criterion in Figure S1 were re-clustered by incorporating information from corresponding light-chain data (altered criterion described under *Light chain analysis*).

**
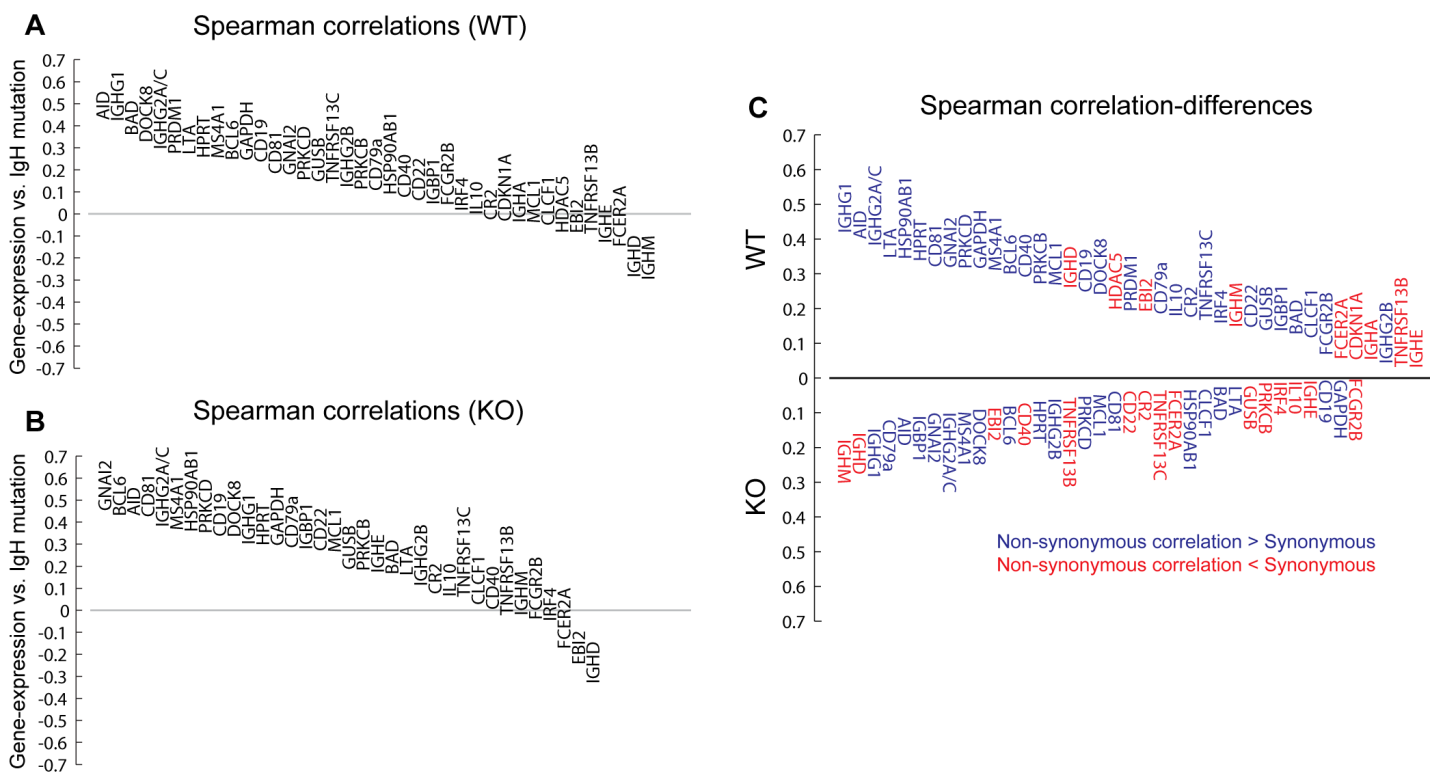
**

**Figure S6: Gene-expression correlations calculated by equal-sampling of B-cell clonal lineages (with VL-correction of lineage-identities).** Spearman correlations previously calculated across all cells (Figure 2, Figure S3) between heavy-chain (IgH) mutations and gene expression were re-calculated by averaging correlations obtained by iteratively and randomly sampling cells from each lineage (with abundances histogrammed in Figure 5). Genes which are positive in only a subset of cells from each lineage (so that in at least one instance of random sampling they are all negative) are excluded from the plot.

**
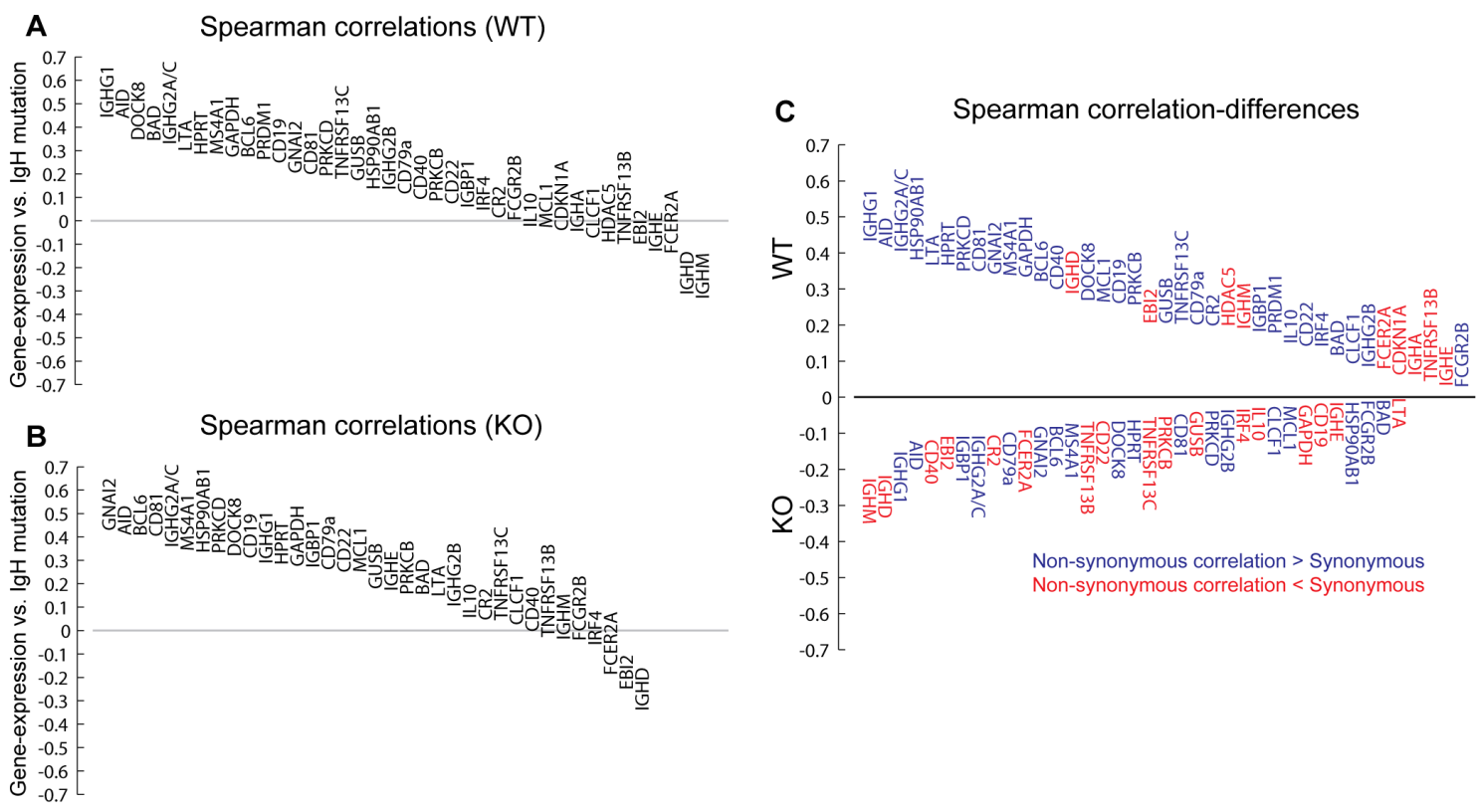
**

**Figure S7: Gene-expression correlations calculated by equal-sampling of B-cell clonal lineages (without VL-correction of lineage-identities).** Equal-sampling of mutation/gene-expression pairs applied to clonal lineages that are assigned by heavy-chain CDR3 similarity alone (as with Figure S1).

**
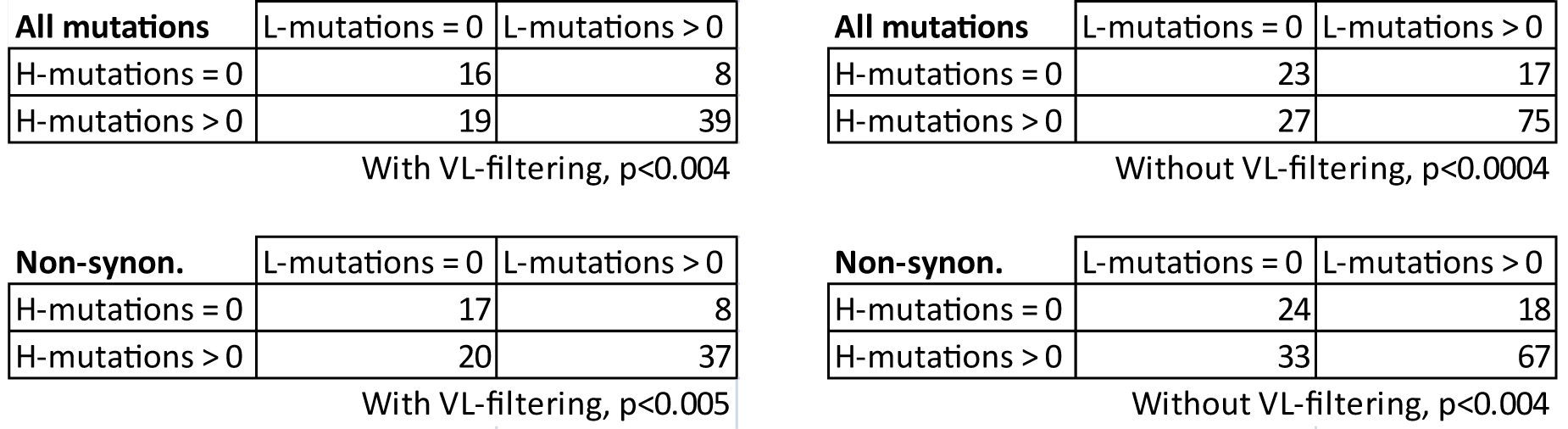
**

**Table S1: Contingency tables for heavy/light chain mutation with and without stringent light-chain filtering.** P-values are calculated using a one-tailed Fisher’s Exact Test to quantify the degree to which an un-mutated or mutated heavy chain predicts an un-mutated or mutated light-chain, respectively.

**Table S2**: **Spearman permutation p-values (two-tailed) derived from the lineage-sampled mutation-correlation data plotted in Figure 6 (with VL-correction of lineage-identities).** P-values are averaged across 10^5^ trials in which cells from each clonal lineage are sampled once. Genes which are positive in only a subset of cells from each lineage (so that in at least one instance of random sampling they are all negative) are excluded from the list.

**Table S3**: **Spearman permutation p-values (two-tailed) derived from the lineage-sampled mutation-correlation data plotted in Figure S7 (without VL-correction of lineage-identities).** P-values are averaged across 10^5^ trials in which cells from each clonal lineage are sampled once. Genes which are positive in only a subset of cells from each lineage (so that in at least one instance of random sampling they are all negative) are excluded from the list.

**Table S4: Sequencing-amplicon primer-sets.** Outer forward primer-sets VHF-1 and VLF-1 (adapted from Novagen cat. 69831-3) were used during pre-amplification of sequencing-amplicons. Inner forward primer-sets inVH and inVL (adapted from Rohatgi et al 2008) were used independently during post-amplification (prior to sequencing). Reverse primer-sets VHR-seq and VLR-seq targeting constant-regions were used during both steps. Fractions in left-most columns denote proportions of primers in mixes (all add to 1).

**Table S5: EvaGreen PCR assay primer-sets**. Assays were designed by Fluidigm DELTAgene. Antibody isotype-specific assays (IgHA, IgHD, IgHE, IgHG, and IgHM) were designed against portions of consensus sequences of mouse CH1 regions available on the IMGT database that were mutually exclusive to sequencing amplicons. IgHG2A/C primers were designed against both IgHG2A and IgHG2C subclasses, due to homology. Only those genes producing positive signal in at least one cell are shown in Figure 1 of the main text.

**Table S6: Summary of gene biochemical features, roles in B cell biology, and chromosomal locations.** See main text for references.

**References**

Jiang N, Weinstein JA, Penland L, White RA 3rd, Fisher DS, Quake SR (2011) Determinism and stochasticity during maturation of the zebrafish antibody repertoire. Proc Natl Acad Sci U S A108:5348-53.

Kabat E A, Wu T T, Perry H M, Gottesman K S, Foeller C (1991) Sequences of Proteins of Immunological Interest. Vol. 1. 5th ed, 1991.

Rohatgi S, Ganju P, Sehgal D (2008) Systematic design and testing of nested (RT-)PCR primers for specific amplification of mouse rearranged/expressed immunoglobulin variable region genes from small number of B cells. J Immunol Methods. 339:205-19.

1. Accuracy calculations assumed all genes in positive control were positive, including those such as RAG1 which were almost certainly true-negatives. Accuracy percentages should therefore be considered lower-bounds. [↑](#footnote-ref-1)
